# Exploration and Analysis of the Aesthetic Cognitive Schema of Contemporary Western Urban Landscapes

**DOI:** 10.3390/ijerph18105152

**Published:** 2021-05-13

**Authors:** Shan Gao, Songfu Liu

**Affiliations:** Key Laboratory of Cold Region Urban and Rural Human Settlement Environment Science and Technology, Ministry of Industry and Information Technology, Department Architecture Landscape, School of Architecture, Harbin Institute of Technology, Harbin 150000, China; liusongfu_hit@126.com

**Keywords:** urban landscape, aesthetic perception, cognitive schema, qualitative analysis

## Abstract

The multidimensional iterative composition of urban landscapes and the formation mechanism of the aesthetic perception dimension are elucidated. The cognitive schema theory aims to reveal the intrinsic mechanism of urban landscape aesthetic activities. Using London as an empirical case to explore the representation and structure of urban landscape aesthetic, a cognitive schema, the cognitive map of its urban landscape, was constructed based on the qualitative analysis of the texts derived from travel notes. Eight aspects of urban landscapes, together with 21 representative concepts of cognitive schema closely related to aesthetic perception, indicate the structures and approaches people perceive in urban landscapes. This article provides experience and reference for urban landscape enhancement and related practices in China by studying the contemporary Western urban landscape.

## 1. Introduction

The urban landscape is the product of human settlement activities, whose essence is the projection of man–earth relationships and social relationships within cities [1], including tangible material elements and intangible connotations of value and significance. This comprehensive cultural phenomenon is an intuitive carrier of urban image characteristics and cultural features and an important medium for people to perceive and experience cities. The nice urban landscape is the basis of collective memory formation in urban residents and condensing “nostalgia,” helping to create the senses of place and identity. Our country’s development model has changed from “high increment” into “high quality,” attracting attention to improving urban residents’ quality of life and the urban landscape. Thus, as an essential part of human settlements, the urban landscape has become one of the key objects of improvement. The notice jointly issued by the Ministry of Housing and Urban-Rural Development and the National Development and Reform Commission in 2020, stressing management on cities and architectural style, reflects the important role of the urban landscape in the aspects of “strengthening cultural confidence” and “embodying urban spirit”.

Urban landscape aesthetic is the basis for the formation of collective memory of urban residents and the cohesion of “homesickness,” and leads to the shaping of a sense of place and identity. “Landscape” means an area, as perceived by people, whose character is the result of the action and interaction of natural and/or human factors [2]. The creation and improvement of urban landscape shall fully consider people’s demand, based on a people-oriented perspective. Such demand is contained in the interaction between human and urban landscape, and the aesthetic activity is the most basic and most representative form of interaction. In Western landscape theory, the definition of “landscape” itself is inseparable from aesthetic perception [3], which indicates a composition based on the fusion of perception dimension and entity dimension [4]. Landscape aesthetic activity refers to human practice activities that use landscape as a medium to create aesthetic value. With the evolution of Western cultural thoughts, the former universal aesthetic standard has gradually disintegrated [5], and the aesthetic paradigm characterized by diversity and the subjective difference has become increasingly prominent. Although aesthetic preferences vary from person to person [6], in an urban landscape’s aesthetic activities, the aspects and contents that people focus on are consistent [7]. In other words, there are common influencing factors in the aesthetic experience of the urban landscape. The analysis and revelation of these common factors will help to understand the urban landscape’s aesthetic activities from structural and disciplinary, giving feedback to urban landscape design and optimization. Therefore, this article introduces psychology’s “schema theory” and, with the help of the cognitive map analysis method, reveals the connotation of the urban landscape’s aesthetic activities in the form of a cognitive schema. Besides, combined with London’s practical case, it analyzes the representation and structure of the urban landscape’s aesthetic cognitive schema to provide reference and basis for related construction in our country by studying contemporary Western urban landscape aesthetic.

The UK has a long history of urban development and a mature urban landscape system, which is of reference significance for the urban landscape system under development in China. This can be explained from two perspectives. On the one hand, the aesthetic activities of urban architectural landscape can be explained effectively by drawing a cognitive map and constructing a cognitive schema. This method has the potential to be applied to different cities in China, and will contribute to the formation of the aesthetic cognitive schema of urban architectural landscape with locality, and help to identify and highlight the unique urban landscape from the internal mechanism level. On the other hand, the representation of aesthetic cognitive schema of urban architectural landscape and the recognition of its corresponding landscape dimensions can be combined with the regulatory detailed planning and urban design guidelines. The corresponding indicators are set for rigid control or elastic control according to the degree of correlation between these key representations and dimensions and urban features. In this way, it helps indicate the direction for the design and optimization of urban architectural landscape, and create a livable urban architectural environment with a sense of place.

## 2. Aesthetic Perception Dimension of the Urban Landscape

The urban landscape scope covers the phenomenon of multiple dimensions from the whole city to the local environment. According to space production theory, the urban landscape has three attributes: spatiality, timeliness, and sociality [8]. On the one hand, natural and artificial factors and their spatial combination constitute the urban landscape’s basic structure. On the other hand, the diachronic characteristics, including seasonal variation and the flow of people and cars in the landscape, create the dynamic landscape characteristics. Also, the urban landscape is shaped and maintained in a specific sociocultural context, which is also a reflection of social values and spiritual culture.

However, there are differences between the subjective perception and the objective world. People’s aesthetic feeling of the urban landscape is not directly derived from the objective environment but filtered by their perception and experience. The Canadian scholar, Julian Smith, found a transformation trend from “Visual-based” to “Experience-based” in the comparison between the modern architectural paradigm in the 20th century and the postmodern architectural paradigm in the 21st century [9]. French scholars represented by Hélène Jannière pointed out that from the architectonic, geological, and botanical perspectives, the essence of urban landscape was a type of “Perceived Entity” on the aesthetic level, integrating the perception of all aspects of the city [10]. The perception was not only visual but also involved various other senses.

**Figure 1:**
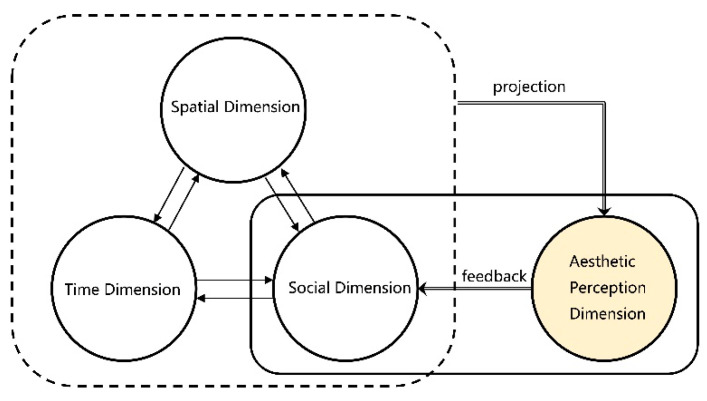
Multidimensional Iterative Relationship of Urban Landscape.

Thus, in addition to the space–time and social dimensions, there is another dimension of the urban landscape: the urban landscape’s aesthetic perception. This aesthetic perception does not exist independently but is linked to other dimensions. It is the projection of objective space–time and society in the thinking of the experiencer. The aesthetic experience’s advantages and disadvantages are usually the motivation for people to change the urban landscape, so the aesthetic perception dimension reacts to the social dimension and is incorporated into the dialectical relationship of multiple iterations in the form of a feedback mechanism (Figure 1).

**Figure 2:**
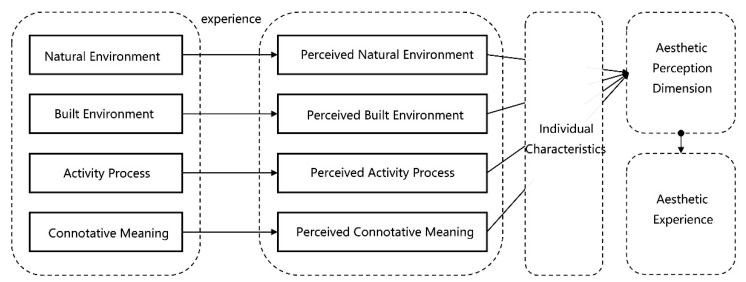
Formation Mechanism of Aesthetic Perception Dimension.

The urban aesthetic theory points out that different space experience is the basis of aesthetic experience [11]. People cannot see the whole picture or understand all the details when experiencing the urban landscape. Instead, the information is obtain as fragmented perceptions from different angles, and then by the processing of individual characteristics (including personality, experience, and memory), they finally construct the overall perception dimension in their mind [12], producing the corresponding aesthetic feeling (Figure 2). Therefore, the overall aesthetic perception dimension consists of local perception, which depends on different local space experience types. These specific types of space experience are the way of urban landscape perception. The exploration of the existing structure and mode is the starting point to study the aesthetic perception dimension.

## 3. Aesthetic Perception Dimension of the Urban Landscape

It can be seen from the previous text that the aesthetic perception dimension is the mapping constructed in people’s minds after experiencing the urban landscape, so the deconstruction of this dimension should be based on its deep psychological mechanism. In the field of psychology, environmental information is usually presented in people’s thinking in the form of “Cognitive Schema”. 

The concept of “schema” was first put forward by the German philosopher Immanuel Kant, referring to the link between the thinking and perception entities [13]. Later, Swiss scholar Jean Piaget, British scholar Frederick Bartlett, and American scholar Richard Anderson introduced the term into psychological research and gradually developed it into a systematic theory, namely Schema Theory. Schema Theory is also called the Schema-based Theory for the word schema, which can be understood as the basic information organization unit in the cognitive process. 

The Schema Theory points out that the cognitive subject understands and interprets the world by cognitive schema [14]. The schema is the system framework of cognitive activities and the organization form of perception information, reflecting people’s structured cognition formed on the cognitive objects [15]. From the perspective of schema theory, when people perceive aesthetics, they first aggregate the scattered information they perceive into several cognitive schemas and then establish connections with their known knowledge and experience. After comparing the existing schema, they classify them into the existing schema through assimilation or construct the new schema through accommodation. 

As the deep cognitive framework of aesthetic activities, the cognitive schema is abstract and plays a role in the “mechanism.” Based on this, an American scholar, Edward Tolman, put forward Cognitive Mapping [16]. The cognitive map is the domination of cognitive schema, representing perception dimensions (perception results and thinking impression) constructed by cognitive subject in mind for the external environment. This representation focuses on the differences and relationships between the perception objects, which are screened, organized, and endowed with meaning by the cognitive subject. The cognitive map includes the two basic types of conceptual representation and spatial representation, reflecting a particular experience’s structure and composition. The conceptual cognitive map is composed of concept words (variables), connecting lines and labels. It is an abstract representation of the cognitive hierarchy and structural relationship of a certain problem. The typical representative is the narrative structure map drawn by American artist Mark Lombardi [17]. The spatial cognitive map is similar to the actual map, and the difference is that it highlights the focus of the perception and omits many other details. In his city image research, Kevin Lynch used this kind of cognitive map [18]. There are differences in the representation of cognitive schema between the conceptual schema and the spatial schema. The former represents the internal structure, and the latter is closer to the representation of cognitive results. This article chooses a conceptual cognitive map as its representation approach to launch the analysis to deconstruct the urban landscape’s aesthetic cognitive schema from the structure and dimension.

## 4. Exploration of the Representation of Aesthetic Cognitive Schema of the Urban Landscape

### 4.1. Spatial Layout

#### 4.1.1. Introduction to the Research Area

Western countries have researched urban landscape earlier as a long-term research foundation and formed many excellent cases, among which Britain is the most typical. Britain is the first country to attach policy guidance to the urban landscape’s quality [19]. It has put forward the management policies of Strategic Views and Protected Views [20] and Landscape Character Assessment and Historic Townscape Characterization [21], which has provided us with good references to interpret and optimize the urban landscape. The above policies and methods have reflected the attention to the aesthetic level of the urban landscape. 

London as a capital city is located in the southeast of Britain and seated on both sides of the Thames River, which affects the world in politics, economy, culture, traffic, and art. Due to the centrality in many aspects and the diversity in the cultural phenomenon and natural ecology, London has created diverse urban landscape types and an integrated urban landscape system. Therefore, taking London as the research area, we can obtain more comprehensive landscape perception and experience materials than other cities to analyze the urban landscape’s aesthetic cognitive schema.

#### 4.1.2. Selection of Research Materials

There are two trends in the past research on landscape aesthetics: one is theoretical speculation, the other is the evaluation based on questionnaire and scale. Although these two kinds of research help us comprehend the psychological basis of aesthetics and understand the aesthetic value of specific objects, they have obvious defects in the description of perceptual details and situational content interpretation [22]. In recent years, the rising “qualitative research” takes phenomenology as the philosophical basis and methodology. Guided by the interpretation of connotation and meaning, it applies to aesthetic research involving comprehensive experience and focusing on meaning generation. 

The qualitative research features by Qualitative Data Analysis, stressing that the attributes and characteristics are carrying the value and meaning rather than the quantitative neural value. The qualitative data have a wide range of types. Any phenomenon with cultural attributes is the embodiment of people’s thoughts and ideas. Therefore, social facts such as texts, pictures, and objects can be used as research materials. Among them, urban travel notes are textual qualitative data formed after people experience a certain city, such as people walking tracing, gate counting, etc... Travel notes record the experience of the interaction between the experiencer and the landscape, in which the processes of aesthetic activities and the results of aesthetic cognition are reflected. Different travel notes involve the aesthetic experience of different cognitive subjects. Although there are differences in individual preferences, as mentioned above, there are similarities in cognitive focus and deep structure, and this regularity is the key to our deconstruction of the aesthetic cognitive schema of the urban landscape.

In summary, this article takes London as the research area, adopts qualitative research methods, and takes travel texts in weblogs as the research material to explore the cognitive schema of urban landscape aesthetics. The specific research mainly searches and obtains travel notes about London from domestic websites such as Mafengwo, Sina Blog, Bai Jiahao, Qiong You, and Jianshu. After screening, it retains 56 travel notes about London city as the analysis object. These travel notes will be used to explore the cognitive structure of urban landscape aesthetics. Specifically, by analyzing the correlation model between concepts, they construct the cognitive map and reveal the internal cognitive schema structure.

### 4.2. Concept Coding and Cognitive Map Construction

Decision Explorer (DE) (universities of Strathclyde, Glasgow, Scothland) is a new developed software aiming at social issue— the qualitative information that surrounds complex or uncertain situations. This article adopts DE (Version 3.5.0) for analysis of qualitative data. The DE could organize people’s thinking by capturing ideas and the relationships between them, making the interdependencies explicit. This software is an effective tool for studying the conceptual cognitive map, creating variables, constructing a cognitive map based on the text data, and analyzing and interpreting the structure and relationship between variables. 

**Figure 3:**
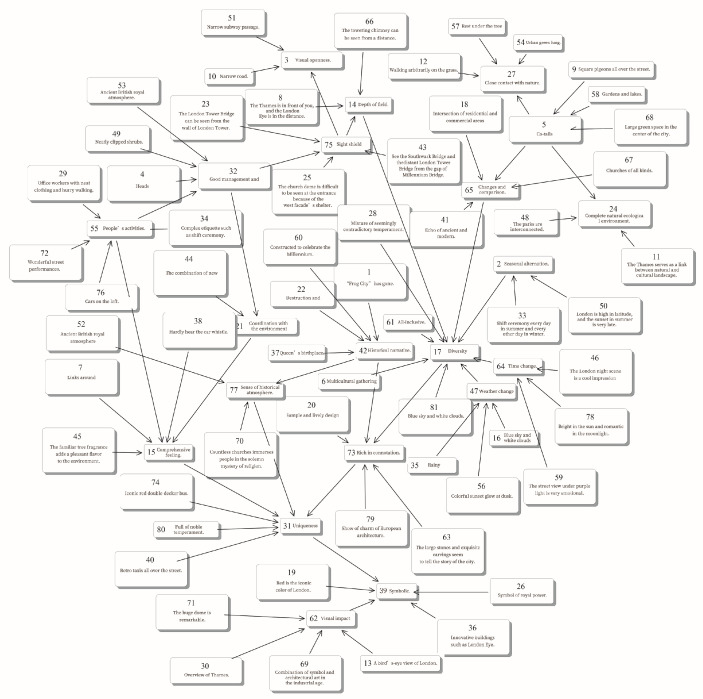
Cognitive Map of Urban Landscape Aesthetics of London (Output by Decision Explorer).

Before constructing the cognitive map, it preprocessed the 56 travel notes and coded the representation aesthetic perception texts. Inputs actually include two aspects: one is the variables filtered from the travel notes, and the other is the correlation between the variables. The coding concepts were then organized in the form of “Variables.” The coding process also involves combining similar concepts: the integration of different texts expressing the same aesthetic perception phenomenon, to avoid the bias of model results caused by redundant information. Finally, 81 variables were coded and entered into the software platform to construct London’s conceptual cognitive map of urban landscape aesthetics (Figure 3). The directional line among the variables represents the relationship among various phenomena, including the three basic types: Causal, Temporal, and Connotative.

**Figure 4:**
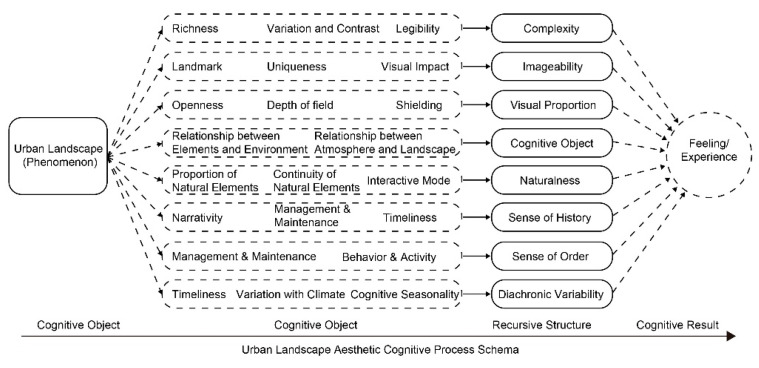
Function of Representation and Structure of Urban Landscape Aesthetic Cognitive Schema in Cognitive Activities.

According to the above analysis, the aesthetic activity of urban landscape is carried out with the assistance of cognitive schema, and the urban landscape phenomenon is abstracted as schema representation in the mind of the experiencer, and then recursed to a higher-level schema structure to form the final feeling (Figure 4). On the whole, people’s cognition of urban landscape is closely related to cognitive schema representations. In terms of its structure, these cognitive schemas belong to eight dimensions of urban landscape: complexity, imageability, visual proportion, coordination, naturalness, sense of history, sense of order, and diachronic variability.

## 5. Exploration of the Representation of Aesthetic Cognitive Schema of the Urban Landscape

The above cognitive map is the presentation of the relationship among the aesthetic perception phenomenon, and the key purpose of constructing a cognitive map is to reveal its internal structure. For the phenomenon with the function of hub and medium in the whole cognitive map structure, its function is similar to the cognitive activities’ schema mechanism. It is a concrete representation of abstract schema in thinking. 

The conceptual cognitive map interpretation shall first start with the types of variables and clarify their basic components. As shown in Table 1, the types of variables in the London urban landscape’s aesthetic cognitive map are mainly divided into Heads, Tails, and Composite Tails, which are Co-tails in short. The head in the Heads refers to the arrow end of the relational line, while the Heads refer to the relational line variables’ tail. There is no relational line in the cognitive map starting from these kinds of variables; that is, the perceptual phenomenon represented does not lead to other phenomena. For the Tails variable, the opposite is true; the perceptual phenomenon represented by the above variables is only the “cause” of other perceptual phenomena. Many relation lines are led out from the Co-tails, representing the variables that may trigger various other perceptual phenomena. Another characteristic index helpful to understand its basic component is the number of “Loop,” referring to the fact that multiple variables are cyclically connected to form a closed loop. It needs to recheck the model if there are many loops.

After mastering a cognitive map’s basic constitution, this article further analyzed its internal structure, including Domain Analysis, Central Analysis, Cluster Analysis, and Potency Analysis. Results suggest that the Domain Analysis and Central Analysis are the most effective methods to reveal the key variables (see Table 2 for the analysis results). Both the Domain Analysis and the Central Analysis focus on the “connectivity” of variables, whose purpose is to reveal many “busy” nodes connecting in and out in the model. However, the former focuses on the number of direct connections in a single level from the variable to the outside. Simultaneously, the latter brings more indirect connections into the analysis scope by way of circle weight.

After mastering a cognitive map’s basic constitution, this article further analyzed its internal structure, including Domain Analysis, Central Analysis, Cluster Analysis, and Potency Analysis. Results suggest that the Domain Analysis and Central Analysis are the most effective methods to reveal the key variables (see Table 2 for the analysis results). Both the Domain Analysis and the Central Analysis focus on the “connectivity” of variables, whose purpose is to reveal many “busy” nodes connecting in and out in the model. However, the former focuses on the number of direct connections in a single level from the variable to the outside. Simultaneously, the latter brings more indirect connections into the analysis scope by way of circle weight. 

It can be seen from the schema theory that aesthetic cognition is a recursive process from “perception phenomenon” to “thinking schema,” so “schema” is the abstraction and integration of phenomenon to some extent. Therefore, in the conceptual cognitive map, the heads in the result end of the relational network, the variables with a high correlation degree in domain analysis, and variables with higher central analysis scores possess the conditions to become the schema’s representation. Moreover, the comprehensive judgment on urban landscape’s aesthetic cognitive schema can be made from these three aspects. 

Based on the above analysis, 21 concepts with high centrality are recognized from the 81 aesthetic perception phenomena, the key links in aesthetic activities. On the one hand, many other aesthetic perceptions need to be formed through these nodal concepts. On the other hand, these nodes are the intermediate media for the perception phenomena to be internalized into feelings. In a previous text when people perceived aesthetics, they first aggregated the scattered information they perceived into several cognitive schemas, and then constructed aesthetic experience in the comparison process with the existing schema. Therefore, these 21 concepts with centrality are starting points for people to understand and interpret the urban landscape, more precisely, the representations of the cognitive map of urban landscape aesthetics. After translating the individual colloquial expressions into the representation schema concepts, these concepts are further aggregated into eight dimensions of urban landscape aesthetic cognition based on the connotation (Table 3).

The complexity, imageability, visual scale, coherence, and naturalness belong to the tangible dimensions of the urban landscape, and the related aesthetic cognitive schema reflects the subjects’ attention to the external aspects of the urban landscape: too complex information type and information amount will cause the loss of legibility of urban landscape, while too low complexity will lead to monotony and boredom. The imageability refers to the urban landscape’s external characteristics as having unique identifiability, which may stimulate the observer to form a clear picture in his mind, to arouse the aesthetic interest, and leave a deep impression after the experience. The angle of view and the field of vision will create different aesthetic situations. The coherence means the integrality and continuity perception consistent with the background environment, and the interference from the visual and auditory levels is the biggest threat to this experience. Meanwhile, Biophilia makes people love to contact with the natural environment and establish a relationship with it, and except for its ecological functions, natural elements in the urban landscape have played the role of Restorative Environment, which restores people’s energy and thinking efficiency in the process of aesthetic experience. On the other hand, historicity means orderliness, representing the aesthetic subjects’ attention to the urban landscape’s intangible dimension: the landscape is the carrier of meaning and value, which is narrative in itself, and the overall historical environment can create an aesthetic conception with rich historicity. The external orderliness is the embodiment of the intangible connotation of the system, management, and culture. The orderliness of the urban landscape forms the corresponding schema within the mind of the experiencer and collides with the familiar existing schema to generate the aesthetic experience of the implication level. In addition, the ephemera of the urban landscape is one of the key dimensions of its aesthetics. The instant dynamic change breaks the existing style and changes people’s activities in the landscape, thus creating a novel and pleasing aesthetic experience.

## 6. Conclusions

The urban landscape is the carrier of urban characteristics and cultural features and the critical medium for people to perceive and experience the city. People interact with the urban landscape through aesthetics, while, in fact, the aesthetic activity is a process of comprehensive spatial experience. The perceived urban landscape constructs the aesthetic perception dimension within the experiencer’s mind to be as the projection of the objective space–time and society in thinking. Cognitive schema is the deep structure of cognitive activities. The subjects understand and interpret the world relying on the schema. The aesthetic feeling is also formed in the process of assimilation and adaptation of cognitive schema. By analyzing the qualitative data, constructing the cognitive map, and exploring the relationship among the perception phenomenon, it can deconstruct the urban landscape’s aesthetic perception dimensions and identify the urban landscape dimensions closely related to the aesthetic perception and the corresponding schema representations. These dimensions and schema reveal people’s way of looking at the urban landscape and the cognitive structure and point out the direction of the design and optimization of the urban landscape so as to create a local and livable urban environment. For cities in different regions and different cultural circles, the architectural landscape environment may be different in element composition, key features and perception approaches. Therefore, the conclusion of this study on London is not universal and should be appropriately quoted and used for reference in the construction of Chinese cities.

## Figures and Tables

**Table 1:** Construction Types of Variables of Perception Phenomenon in Cognitive Map. (Total Number of Variables: 81).

Item	Specific Content		
Heads (4)	3. Visual openness. 39. Symbolic.	24. Complete natural ecological environment.	27. Close contact with nature.
Tail (60)	1. “Frog City” has gone. 4. No cars parked around the street. 6. Multicultural gathering. 7. The sound of Big Ben telling the time is clear and beautiful. 8. The Thames is in front of you, and the London Eye is in the distance. 9. Square pigeons all over the street. 10. Narrow road. 11. The Thames serves as a link between natural and cultural landscape. 12. Walking arbitrarily on the grass. 13. A bird’s-eye view of London. 16. Blue sky and white clouds. 18. Intersection of residential and commercial areas. 19. Red is the iconic color of London. 20. Sample and lively design. 22. Destruction and reconstruction several times. 23. The London Tower Bridge can be seen from the wall of London Tower. 25. The church dome is difficult to be seen at the entrance because of the west facade’s shelter. 26. Symbol of royal power. 28. Mixture of seemingly contradictory temperament. 29. Office workers with neat clothing and hurry-walking. 30. Overview of Thames.	33. Shift ceremony every day in summer and every other day in winter. 34. Complex etiquette such as shift ceremony. 35. Rainy. 36. Innovative buildings such as London Eye. 37. Queen’s birthplace. 38. Hardly hear the car whistle. 40. Retro taxis all over the street. 41. Echo of ancient and modern. 43. See the Southwark Bridge and the distant London Tower Bridge from the gap of Millennium Bridge. 44. The combination of new architecture and the sense of history is not abrupt. 45. The familiar tree fragrance adds a pleasant flavor to the environment. 46. The London night scene is a cool impression. 48. The parks are interconnected. 49. Neatly clipped shrubs. 50. London is high in latitude, and the sunset in summer is very late. 51. Narrow subway passage. 52. Ancient British royal atmosphere. 53. Ancient British royal atmosphere. 54. Urban green lung.	56. Colorful sunset glow at dusk. 57. Rest under the tree. 58. Gardens and lakes. 59. The street view under purple light is very emotional. 60. Constructed to celebrate the Millennium. 61. All-inclusive. 63. The large stones and exquisite carvings seem to tell the story of the city. 66. The towering chimney can be seen from a distance. 67. Churches of all kinds. 68. Large green space in the center of the city. 69. Combination of symbol and architectural art in the industrial age. 70. Countless churches immerse people in the solemn mystery of religion. 71. The huge dome is remarkable. 72. Wonderful street performances. 74. Iconic red double-decker bus. 76. Cars on the left. 78. Bright in the sun and romantic in the moonlight. 79. Show of charm of European architecture. 80. Full of noble temperament. 81. Magnificence and luxurious decoration.
Co-tails (5)	5. Many animals, plants, and water bodies. 32. Good management and maintenance.	42. Historical narrative. 55. People’s activities.	75. Sight shield.
Loops	0		

**Table 2:** Analysis Results of Structural Relationship among Aesthetic Perception Phenomenon.

Analysis Type	Analysis Results	
Domain Analysis	10. Links around 17. Diversity 7. Links around 31. Uniqueness 6. Links around 5. Many animals, plants, and water bodies. 15. Comprehensive feeling. 32. Good management and maintenance. 42. Historical narrative. 55. People’s activities. 73. Rich in connotation. 75. Sight shield.	5. Links around 39. Symbolic. 62. Visual impact. 65. Changes and comparison. 4. Links around 14. Depth of field. 27. Close contact with nature. 47. Weather change. 64. Time change. 77. Sense of historical atmosphere. 3. Links around 2. Seasonal alternation. 3. Visual openness. 21. Coordination with the environment. 24. Complete natural ecological environment.
Central Analysis	17. Rich and diverse—26 from 50 concepts 73. Rich connotation—23 from 51 concepts 31. Uniqueness—22 from 46 concepts 65. Change and contrast—19 from 40 concepts 14. Depth of field—19 from 42 concepts 15. Comprehensive feelings—17 from 31 concepts 75. Sight shield—16 from 31 concepts 55. People’s activities—14 from 27 concepts 39. Symbolic—14 from 27 concepts	32. Management and maintenance—14 from 26 concepts 77. Sense of historic atmosphere—13 from 27 concepts 64. Time change—13 from 30 concepts 47. Weather change—13 from 30 concepts 42. Historic narrative—13 from 27 concepts 5. Many animals, plants, and water bodies—13 from 24 concepts 2. Seasonal alternation—13 from 30 concepts 21. Coordination with the environment—12 from 27 concepts

**Table 3:** Structure of Cognitive Schema of Urban Landscape.

Open Coding	Axial Coding	
Aesthetic Perception Phenomenon	Schema representation	Urban landscape dimension
Multicultural gathering; a mixture of seemingly contradictory temperament; magnificence and luxurious decoration; all-inclusive.	Richness	Complexity
Various churches; echo of ancient and modern; at the intersection of residential and commercial areas;	Change and contrast	
Show the charm of European architecture; huge stones and exquisite carvings seem to tell the story of city; simple and lively design;	Readability	
Innovative buildings such as the London Eye; a symbol of royal power; red is the symbol of London;	Symbolic	Imageability
Full of noble temperament; the red double-decker bus is one of the symbols of London; taxis are also the symbol of London; retro cars around the street;	Uniqueness	
A bird’s-eye view of London; overview of Thames; the huge dome is remarkable; the thick steel cable, combination of the symbol of the industrial age and architectural art;	Visual impact	
Narrow road; narrow subway passage;	Openness degree	Visual Scale
The Thames is in front of you, and the London Eye is in the distance; the towering chimneys can be seen from a distance;	Depth of field	
The high dome cannot be seen from the entrance of the church because of the high west facade; see the Southwark Bridge and the distant London Tower Bridge from the gap of Millennium Bridge; the Tower Bridge can be seen from the wall of London Tower;	Shield	
The combination of the new architecture and the sense of history is not abrupt;	Relationship between elements and environment	Coherence
The familiar tree fragrance adds a kind of flavor to the environment; hardly hear the car whistle; the sound of Big Ben telling the time is clear and beautiful;	Relationship between atmosphere and landscape	
Gardens and lakes; square pigeons all over the street; the large area of green space in the center of the city;	Proportion of natural elements	Naturalness
The parks are interconnected; the Thames River serves as a link to connecting the natural and cultural scenery;	Continuity of natural elements	
Urban green lung; walk arbitrarily on the grass; rest under the tree;	Interactive mode	
The “Fog City” has long been gone; constructed to celebrate the Millennium; Queen’s birthplace; destruction and reconstruction of several times;	Narrative	Historicity
Countless churches make people immersed in the solemn mystery of religion; ancient British royal atmosphere;	Sense of atmosphere	
Neatly clipped shrubs; clean and tidy roads; no cars parked on the street;	Management and maintenance	Orderliness
Cars on the left; excellent street performances; office workers with neat clothing and hurry-walking; complex etiquette of shift ceremony;	Behavioral activities	
The cool London in the night scene is the London in the impression; the street view under the purple light is very emotional; bright in the sun and romantic in the moonlight;	Timeliness	Ephemera
Rainy; blue sky and white clouds; colorful sunset glow at dusk;	Climatic	
Shift ceremony every day in summer and every other day in winter; London is high in latitude, and the sunset is very late in summer;	Seasonality	

## Data Availability

Data available in a publicly accessible repository that does not issue DOIs Publicly available datasets were analyzed in this study. This data can be found here: [http://www.mafengwo.cn// (accessed on 22 March 2021), http://blog.sina.com.cn/ (accessed on 22 March 2021), https://www.qyer.com/ (accessed on 22 March 2021)].
